# Targeting EGFR and Monitoring Tumorigenesis of Human Lung Cancer Cells In Vitro and In Vivo Using Nanodiamond-Conjugated Specific EGFR Antibody

**DOI:** 10.3390/pharmaceutics15010111

**Published:** 2022-12-28

**Authors:** Yu-Wei Lin, Hung-Cheng Su, Emmanuel Naveen Raj, Kuang-Kai Liu, Chien-Jen Chang, Tzu-Chia Hsu, Po-Yun Cheng, Rou-Hsin Wang, Yen-Her Lai, Chien-Hung Chen, Yen-Cheng Lin, Jui-I Chao

**Affiliations:** 1 Department of Biological Science and Technology, National Yang Ming Chiao Tung University, Hsinchu 30068, Taiwan; 2 Institute of Molecular Medicine and Bioengineering, National Yang Ming Chiao Tung University, Hsinchu 30068, Taiwan; 3 Department of Internal Medicine, Hsin-Chu Branch, National Taiwan University Hospital, Hsinchu 30068, Taiwan; 4 Center for Intelligent Drug Systems and Smart Bio-Devices, National Yang Ming Chiao Tung University, Hsinchu 30068, Taiwan

**Keywords:** nanodiamond, cetuximab, lung cancer therapy, EGFR, tumorigenesis

## Abstract

Nanoprobes provide advantages for real-time monitoring of tumor markers and tumorigenesis during cancer progression and development. Epidermal growth factor receptor (EGFR) is a key protein that plays crucial roles for tumorigenesis and cancer therapy of lung cancers. Here, we show a carbon-based nanoprobe, nanodiamond (ND), which can be applied for targeting EGFR and monitoring tumorigenesis of human lung cancer cells in vitro and in vivo. The optimal fluorescent intensities of ND particles were observed in the human lung cancer cells and nude mice under in vivo imaging system. The fluorescence signal of ND particles can be real-time detected in the xenografted human lung tumor formation of nude mice. Moreover, the ND-conjugated specific EGFR antibody cetuximab (Cet) can track the location and distribution of EGFR proteins of lung cancer cells in vitro and in vivo. ND-Cet treatment increased cellular uptake ability of nanocomposites in the EGFR-expressed cells but not in the EGFR-negative lung cancer cells. Interestingly, single ND-Cet complex can be directly observed on the protein G bead by immunoprecipitation and confocal microscopy. Besides, the EGFR proteins were transported to lysosomes for degradation. Together, this study demonstrates that ND-conjugated Cet can apply for targeting EGFR and monitoring tumorigenesis during lung cancer progression and therapy.

## 1. Introduction

Nanoprobes provide a promising strategy for targeted imaging, monitoring of tumors, and cancer metastasis [1,2,3,4]. Fluorescent nanoprobes with various sizes were imaged and correlated with the molecular transport of fluorescent molecules [5]. For instance, a peptide-based four-color fluorescent polydopamine nanoprobe for multiplexed sensing and imaging of tumor-related proteases in living cells [6].

Different types of nanoparticles have been widely used in cancer detection and diagnosis, such as metal [7], non-metal [8], polymer [9], etc. Nanodiamond (ND) is a carbon-based nanomaterial for bio-imaging and bio-sensing [10,11,12]. The nitrogen-vacancy NV centers existing in ND explain the emission of fluorescence [13,14]. ND has been used for labeling and tracking neuronal differentiation and neuronal cells [10]. Furthermore, the long-term photo-stability without photo-bleaching enables ND to be an efficient candidate for bio-imaging [15]. Moreover, ND can be used as a novel nanomaterial for cargos delivery, such as nuclei acid [16] and small molecule drugs [17], since the surface of ND can be functionalized by non-covalent and covalent methods [15,18]. In non-covalent surface modification, ND has been treated with acid to acquire many oxygen-containing functional groups, creating electrostatic bonds, hydrogen bonds, and Van der Waal forces for proteins [17,19,20]. In the case of covalent surface modifications, which based on carboxylated ND, further, can be modified with amines, esters, and other linkers for interested molecules [21].

The epidermal growth factor receptor (EGFR), a member of the ErbB family, has an extracellular ligand binding domain, a transmembrane domain, and an intracellular tyrosine kinase domain [22,23]. EGFR is an ideal candidate for cancer target as it is often overexpressed in cancer cells and the overexpression is associated with advanced disease and poor prognosis. The EGFR-targeted strategy by humanized monoclonal antibodies has been developed against the extracellular domain of EGFR such as Cetuximab (Cet) [24,25]. Cet is a human/murine chimeric monoclonal antibody with an immunoglobulin G1 Fc isotype [24]. It can bind the extracellular domain of EGFR thateffectively blocks the ligand binding and structurally hinder the binding of EGFR to other HER family members [26]. Cet works by promoting the internalization and degradation of the EGFR and destroys the downstream signaling cascades [24]. In non-small cell lung cancer (NSCLC), the Cet resistance is due to the dysregulation of oncogenic shift, explaining that the resistant NSCLC rely on the activation of various receptor tyrosine kinases and their subsequent hetero-dimerization with EGFR to bypass Cet blockage [22,27]. Besides, nuclear EGFR contributes to acquiring resistance to Cet [28].

In this study, the optimal fluorescent intensity of ND particles is determined in the human lung cancer cells and nude mice under in vivo imaging system. The fluorescence signal of ND particles can be real-time detected in the xenografted human lung tumor formation of nude mice. ND-conjugated specific EGFR antibody can apply for targeting EGFR and monitoring tumorigenesis during lung cancer progression and therapy. Tracking of a single EGFR molecule by ND-Cet will provide for understating the function and regulation of EGFR in human lung cancer.

## 2. Materials and Methods

### 2.1. Cell Lines and Cell Culture

The A549 (ATCC number: CCL-185) cell line was derived from the lung adenocarcinoma of a 58-year-old Caucasian male that expressed wild-type EGFR and with K-Ras mutation (G12S). The A549-luc-C8 is a luciferase expressing cell line derived from the A549 cell line obtained from Caliper Life Sciences (Hopkinton, MA, USA). The H1650 (ATCC number: CRL5883) and H1975 (ATCC number: CRL5908) were derived from lung adenocarcinoma that expressed EGFR L858R/T790M mutation. A549, A549-luc-C8, H1650 and H1975 cells were cultured in RPMI-1640 medium (Gibco, Life Technologies, Grand Island, NY, USA). The A549-ON cell line is derived from A549 lung cancer cells with Oct4 and Nanog expression that was kindly provided by Prof. Shih-Hwa Chiou of National Yang Ming Chiao Tung University (Taipei, TW). The expression of Oct4 and Nanog in A549-ON presented the properties of cancer stem-like cells. A549-ON cells were cultured in DMEM medium (Gibco, Life Technologies, Grand Island, NY, USA). DMEM and RPM1-1640 medium was supplemented with 10% fetal bovine serum (FBS), 100 units/mL penicillin, 100 µg/mL streptomycin and sodium bicarbonate. These cells were cultured at 37 °C and 5% CO_2_ in a humidified incubator (310/Thermo, Forma Scientific, Inc., Marietta, OH, USA).

### 2.2. Chemicals and Antibodies

Hoechst 33258 was purchased from Sigma-Aldrich (St. Louis, MO, USA). Cetuximab (Cet) was from Merck Serono (Darmstadt, DE, USA). Coomassie blue was purchased from Amresco (0472, Solon, OH, USA). BCA protein assay kit was purchased from Piece (23225, Pierce, Rockford, IL, USA). Primary antibodies and working dilution for Western blot analysis used in this study as the follows: anti-EGFR (1/100, #4267p, Cell Signaling, Boston, MA, USA), anti-phospho EGFR Tyr 1068 (1/100, #3777, Cell Signaling), Anti-Nanog (1/1000, #4903, Cell Signaling) anti-actin (1/5000, MAB1501, Millipore, Billerica, MA), anti-BSA (1/1000, B2901, Sigma, St. Louis, MO, USA) antibodies, Anti-Oct4 (1/1000, ab109183, Abcam, Inc. San Francisco, CA, USA). Primary antibodies and working dilution for immunofluorescence staining used in this study as the follows: anti-EGFR (1/100, #4267p, Cell Signaling), anti-LAMP1 (1/100, #328601, Biolegend, San Diego, CA, USA) antibodies. Secondary antibodies and working dilution for Western blot analysis and immunofluorescence staining were used in this study as the follows. Anti-mouse IgG-HRP (sc-2005, Santa Cruz, CA, USA) and anti-rabbit IgG-HRP (sc-2004, Santa Cruz) antibodies were used for Western blot analysis. Anti-rabbit Hilyte 488 (1/100, 61056-H488, AnaSpec, Fremont, CA, USA) anti-mouse Alexa Fluor 405 (1/100, A-31553, Molecular Probes Invitrogen, Carlsbad, CA, USA) were used for immunofluorescence staining.

### 2.3. Preparation and Characterization of ND-Cet

Raw nanodiamond powders of synthetic type 1b diamond powders with an average size of 100 nm were purchased from Element six (Micro+ MDA, Element Six Ltd., Shannon, Ireland) Fluorescent ND particles were purchased from FND Biotech or LuminX Biotech (Taipei, Taiwan). ND (10 µg) was added with varied concentrations of Cet (10 µg, 5 µg, 2.5 µg, 1 µg, 0.5 µg, 0.25 µg) to form ND-Cet pellet, which was used for investigation of the binding affinity, loading efficiency and mixture time. Cet were kindly provided by Dr. Johnson Lin of Mackay Memorial Hospital (Taipei, Taiwan) from Merck Serono (Darmstadt, DE, USA). ND-Cet solution was prepared for storage and other experiments after determination of the proper binding ratio and mixture time. The ND-Cet were prepared by a simple absorption of ND and Cet for 15 min, 4 °C, followed by centrifuge at 12,000 rpm for 10 min, 4 °C, then discard the suspension, which was unbinding Cet. In order to completely remove the unbinding Cet, the pellets were washed with deuterium-depleted water (DDW) twice, and the pellets were finally collected for the following experiments.

### 2.4. Binding Affinity of Cet and ND by SDS-PAGE and BCA Assays

The binding affinity of ND-Cet was analyzed by sodium dodecyl sulfate polyacrylamide gel electrophoresis (SDS-PAGE) analysis. The ratios of NDs and Cet (*w*/*w*) were 1:0.025, 1:0.05, 1:0.1, 1:0.25, 1:0.5, and 1:1 that prepared in DDW for binding affinity analysis. The mixtures of ND and Cet were inverted for 15 min at room temperature. The mixtures were centrifuged at 12,000 rpm for 10 min, and the pellets were washed with DDW twice. After SDS-PAGE electrophoresis, the gels were stained with coomassie blue (0.1% coomassie blue, 10% acetic acid, 20% methanol, and 45% DDW) for 1 h and washed with de-staining buffer (10% acetic acid, 20% methanol, and 70% DDW) for 2 h. The band intensities were quantified for calculating the binding affinity of Cet and ND. In addition, the binding affinity of ND-Cet was further analyzed by bicinchoninic acid (BCA) analysis. The ratio of ND and Cet (*w*/*w*), 10:10 μg and 10:2.5 μg was prepared in DDW for binding analysis. The mixtures of ND and Cet were inverted for 15 min at room temperature. The mixtures were centrifuged at 12,000 rpm for 10 min, and the pellets were washed by DDW twice. The protein concentrations of Cet in the ND-Cet complex were determined by the BCA protein assay kit (23225, Pierce, Rockford, IL, USA). The concentrations of Cet (2, 4, 6, 8 and 10 μg) in 200 μL BCA reagent were used for the standard curve.

### 2.5. Antibody Labeling with Alexa 488

To observe the Cet conjugated with ND, a fluorophore was covalently attached to a small amount of Cet antibody. Approximately 20 μg of Cet were applied into resin to react with fluorophore dye for 2 h at room temperature. After wash and neutralization, the resin was eluted to collect the Cet labeled with a fluorophore. The APEX^TM^ antibody labeling kit was purchased from Invitrogen (A10468, Carlsbad, CA, USA).

### 2.6. Zeta Potential Analysis

Zeta potential was used to measure the surface charge of particles in suspension. To measure Zeta potential, the particles of moving rate were changed and tracked in an electric field. The concentration 0.05 μg/mL of ND and ND-Cet in DDW were ultrasonic for 20 min at room temperature before use. The concentration of 5 μg/mL Cet was dissolved in DDW. The surface charges of ND, ND-Cet, and Cet were analyzed by Zeta potential instrument (Zeta PALS, Brookhaven Instruments Co, Holsville, NY, USA).

### 2.7. Dynamic Light Scattering (DLS) Analysis

To examine the size distribution of ND, Cet, ND-Cet (ND-Cet, 1:0.25, *w*/*w*) by dynamic light scattering (DLS) (BI-200SM, Brookhaven Instruments Co., Holtsville, NY, USA). The concentration of 40 μg/mL ND and ND-Cet complex in DDW was prepared and analyzed.

### 2.8. Scanning Electron Microscopy (SEM) and Transmission Electron Microscopy (TEM)

To examine the morphology and dispersion of ND and ND-Cet, the SEM was performed by scanning electron microscope (SEM) (S6700, JEOL, Tokyo, Japan) for observation. Samples prepared by the concentration of 40 μg/mL of ND and ND-Cet (1:0.25, *w*/*w*) in DDW were mounted on the silica slide, then placed the samples at −80 °C for O/N to be freeze-drying. After drying, the samples were coated with a thin layer of gold by sputtering for 15 s, with a 10-mA current, using an Auto Fine Coaters (JEC-1600, JEOL, Tokyo, Japan). A 1 μg/mL droplet of solution of ND or ND-Cet was dispensed in the carbon-coated copper TEM grid. The solvent was evaporated, and the TEM (HITACHI HT7700) was used to verify the presence of ND or ND-Cet on the carbon-coated copper TEM grid.

### 2.9. Western Blot Analysis

The cells were plated at a density of 7 × 10^5^ cells per 60 mm Petri dish in complete medium for 16–20 h. After the treatment, the cells were lysed in the ice-cold cell extract buffer (pH 7.6) containing 0.5 mM DTT, 0.2 mM EDTA, 20 mM HEPES, 2.5 mM MgCl_2_, 75 mM NaCl, 0.1 mM Na_3_VO_4_, 50 mM NaF, and 0.1% Triton X-100. The protease inhibitors including 1 μg/mL aprotinin, 0.5 μg/mL leupeptin, and 100 μg/mL 4-(2-aminoethyl) benzenesulfonyl fluoride were added to the cell suspension. The lysate was vibrated for 30 min at 4 °C and centrifuged at 10,000 rpm for 10 min. The protein concentrations were determined by the BCA protein assay kit (Pierce, Rockford, IL, USA). The total cellular protein extracts were prepared; they were separated on 8–12% sodium dodecyl sulfate polyacrylamide gels, and electrophoretic transfer of proteins onto polyvinylidene difluoride membranes. The membranes were blocked overnight at 4 °C using blocking buffer (5% non-fat dried milk insolution containing 50 mM Tris/HCl (pH 8.0), 2 mM CaCl_2_, 80 mM sodium chloride, 0.05% Tween 20, and 0.02% sodium azide). The membranes were sequentially hybridized with primary antibody and followed with a horseradish peroxidase-conjugated secondary antibody. The protein bands were visualized with an enhanced chemiluminescence assay (SuperSignal™ West Pico Chemiluminescent Substrate; Life Technologies). To verify equal protein loading and transfer, actin was used as the protein-loading control. The protein intensities of scanned images were semi-quantified by using Un-Scan-It gel software (ver. 6.1; Silk Scientific Inc., Orem, UT, USA).

### 2.10. Immunoprecipitation Assays

The EGFR binding ability was examined by immunoprecipitation assays. The cells were cultured in 100 mm Petri dish at a density of 2 × 10^6^ cells for 16–20 h. Then the cells were lysed in the ice-cold cell extract buffer (pH 7.6) containing 0.5 mM DTT, 0.2 mM EDTA, 20 mM HEPES, 2.5 mM MgCl_2_, 75 mM NaCl, 0.1 mM Na_3_VO_4_, 50 mM NaF, 0.1% Triton X-100. The protease inhibitors including 1 μg/mL aprotinin, 0.5 μg/mL leupeptin, and 100 μg/mL 4-(2-aminoethyl) benenesulfonyl fluoride were added to the cell suspension. The protein concentration was determinateby BCA protein assay kit (23225, Pierce, Rockford, IL). Total protein extracts were prepared. The protein G Sepharose^TM^ (GE Healthcare, Danderyd, Sweden) was gently vortexes that all of the protein G was uniformly suspended. The protein G were placed into 1.5 mL microcentrifuge tubes and washed the protein G with 600 μL immunoprecipitation buffer and centrifuge at 4 °C, 2500 rpm for 5 min, repeating the steps for three times. ND, ND-Cet and Cet were separately added to the cell lysates with incubation at room temperature with continuesmixing for 3 h. Then the Protein G was added to the samples and washed with 600 μL immunoprecipitation buffer and centrifuge at 4 °C, 2500 rpm for 5 min, repeatingthe steps for 7 times. After the last wash, the samples were added the loading buffer and analyzed by Western blot.

### 2.11. Fluorescence Intensity of ND in Cells by Flow Cytometry

A549, A549-ON, and PLC26 cells were plated at a density of 7 × 10^5^ cells per 60 mm Petri dish in a complete medium for 16–20 h. After treatment with Cet (2.5 μg/mL), ND (10 μg/mL), and ND-Cet (10 μg/mL) for 24 h, the cells were washed twice with PBS. The cells were trypsinized and collected by centrifugation at 1500 rpm for 5 min and fixed with 75% alcohol at −20 °C overnight. Thereafter, the cells were collected by centrifugation at 1500 rpm for 5 min. Then, the cell pellets were re-suspension in PBS. To avoid cell aggregation, the cell suspension was filtered through a nylon mesh membrane. Finally, the samples were analyzed by flow cytometry (FACSCalibur, Becton Dickinson, San Jose, CA, USA). The fluorescence of NDs was excited at a wavelength of 488 nm, and the emission was collected by >650 nm signal range (FL-3 H). The fluorescence intensity was quantified using a minimum of 10,000 cells by CellQuest software 6.0 (BD Biosciences).

### 2.12. Immunofluorescence Staining and Confocal Microscopy

The cells were cultured on coverslips, which were kept in a 35 mm Petri dish for 16–20 h before treatment. After treatment with ND or ND-Cet, the cells were washed with isotonic PBS (pH 7.4), and then fixed with 4% paraformaldehyde solution in PBS for 1 h at 37 °C. The coverslips were washed three times with PBS, and non-specific binding sites were blocked in PBS containing 10% FBS and 0.3% Triton X-100 for 1 h. Subsequently, the cells were incubated with specific primary antibodies in PBS containing 10% FBS for overnight at 4 °C. Therefore, the cells were washed three times with 0.3% Triton X-100 in PBS. The cells were incubated with fluorescent secondary antibodies in PBS containing 10% FBS for 2.5 h at 37 °C. The nuclei were stained with Hoechst 33258. The prepared samples were analyzed by Multiphoton and Confocal Microscope System (MCMS) (TCS-SP5-X AOBS, Leica, Mannheim, Germany), TCS SP/SP2 (Leica) confocal laser scanning microscope.

### 2.13. Tumorigenesis of Lung Cancer in Nude Mice

All animal studies and experimental protocols in our manuscript were approved by the Institutional Animal Care and Use Committee (IACUC) of the National Chiao Tung University (Hsinchu, Taiwan) (NCTU-IACUC-108060). We separated the nude mice into three groups: vehicle, A549-luc-C8, and A549-luc-C8 plus ND. The A549-luc-C8 lung cancer cells were plated at a density of 2 × 10^6^ cells per 100 mm Petri dish for 24 h. Then the cells were incubated with 100 nm NDs (50 μg/mL for 24 h). At the end of treatment, the ND-treated cells were trypsinized and counted 1 × 10^7^ cells. After centrifugation and collected cell pellets, the cells were resolved in 1× PBS. Intraperitoneal injection vehicle, A549-luc-C8, and ND-bearing A549-luc-C8 lung cancer cells into BALB/c nude mice. The tumor formation detected by cell luminescence signals was observed by an the IVIS system. The luminescence signals were examined at 5, 10, 15, and 20 days after injection.

### 2.14. Statistical Analysis

Each experiment was repeated at least three times. Data were analyzed by Student’s *t*-test. for one group comparison. In a comparison of multiple groups, data were analyzed by two-way ANOVA with LSD post-tests. A *p*-value of <0.05 was considered statically significant in the experiments.

## 3. Results

### 3.1. Optimal Fluorescence Intensities of ND Particles In Vitro and In Vivo

**Figure 1:**
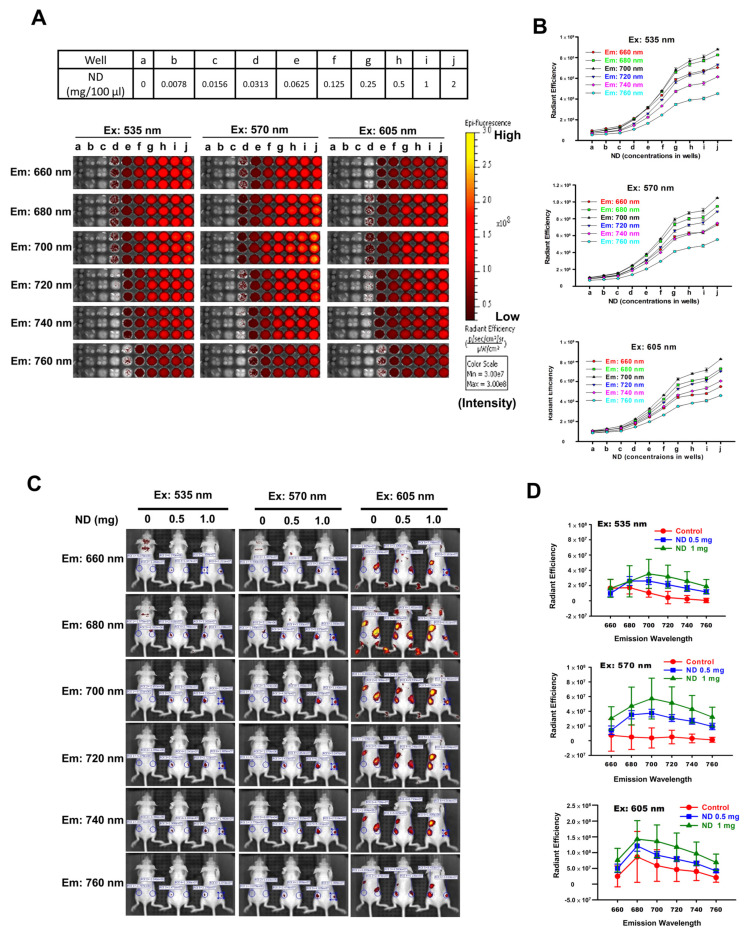
Optimal fluorescence intensities of ND particles in vitro and in vivo under IVIS system. (**A**) Various concentrations of ND particles were loaded in 96-well plates. (a–j) Serial dilutions of the 96-well plates were prepared by mixing 100 μL PBS. IVIS conditions used Bin: 4; FOV: 13.3; f2; 1s. Excitation wavelengths: 350 nm, 570 nm, and 605 nm. Emission wavelength range: 660–760 nm. (**B**) The relative fluorescence signal in ROI region was quantified by IVIS software. (**C**) The fluorescence intensities of ND particles by subcutaneously injection in nude mice. Fluorescent imaging in the anesthetized mouse injected subcutaneous with three nude mice for a group, the left mice injected 100 μL PBS, middle-treated with 0.5 mg/100 μL PBS, and the right mice treated with 1 mg/100 μL PBS. Excitation wavelengths: 350 nm, 570 nm, and 605 nm. Emission wavelength range: 660–760 nm. (Bin: 4, FOV: 13.3, f1, 2s). (**D**) The relative fluorescence signal in ROI region was quantified using Living Image 4.0 software.

To examine the fluorescence intensities of ND particles in vitro and in vivo, ND particles were observed by IVIS analysis. The fluorescence detection of ND samples was carried out by the excitation wavelength at 535, 570, and 605 nm and collected the emission wavelength at 660–760 nm under IVIS. Figure 1A shows the fluorescence signal of serially diluted NDs samples in 96-well plates. We observed optimal fluorescence signal of ND particles of excitation wavelength at 570 nm and emission wavelength at 680–720 nm (Figure 1B). The average radiant efficiencies were increased considerably in a concentration-dependent manner (Figure 1B). Figure 1C shows the in vivo fluorescence images of nude mice after subcutaneous injections of NDs. The images were carried out by exciting at 535, 570, and 605 nm and collecting the emission at 660–760 nm. We found that the fluorescence signal of NDs increased in a concentration-dependent manner. Figure 1D shows that the higher average radiant efficiency of NDs (excitation wavelength at 535, 570, and 605 nm; emission wavelength at 680–760 nm) in the NIR range is in a concentration-dependent manner. NIR imaging is a novel diagnostic technique with potential use in a minimally invasive, nonionizing method for sensitive, and deep tissue imaging [29,30]. These results indicate that the ND fluorescence intensity in the NIR range in vitro and in vivo using IVIS analysis can be used as novel nanoprobe for biomedical applications.

### 3.2. Long-Term Detection of ND Particles In Vivo

**Figure 2:**
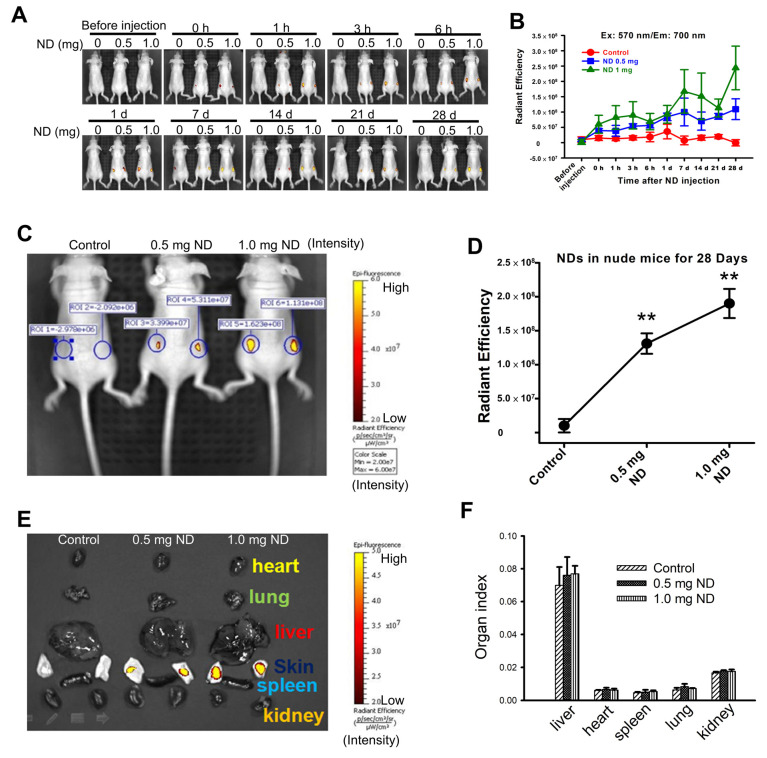
The detection of ND particles in nude mice and organs after 28 days by IVIS system. (**A**) The time-lapse recording of fluorescence intensity of ND particles in nude mice. Fixed wavelength filter in Ex/Em = 570 nm/680 nm and observed the fluorescence signal on different time points by IVIS. Three nude mice for a group, the left mice injected 100 μL PBS, middle-treated with 0.5 mg ND/100 μL PBS, and the right mice treated with 1 mg ND/100 μL PBS. (**B**) The fluorescence intensity of ND−injected nude mice indifferent time point was recorded. The relative fluorescence signal in ROI region was quantified using IVIS software. (**C**,**D**) Representative images of the whole body and quantitation of fluorescent signal in the region. Results were obtained from three separate experiments and the bar represented the mean ± S.E. ** *p* < 0.05 indicates significant differences compared to the control group. (**E**) Representative fluorescence image of organ (from top to bottom are heart, lung, liver, skin, kidney, and spleen) and fluorescence intensities of dissected skin. The skin was imaged immediately following dissection and calculated as fluorescence intensity (photons/second (p/s)). (**F**) Comparison of the organ weight of ND particles injected subcutaneous in different concentrations.

Organic fluorescent probes have been widely used for labeling and tracking of tumors. However, the photobleaching of fluorophores limit the usage on long-term tumor labeling and tracking. ND-contained the fluorophores with NV centers have high photostability and long-lived fluorescence decay lifetime. NDs are an ideal nanoprobe used for labeling and tracking in in vitro and in vivo studies [11,31] However, the long-term detection of ND in vivo needs further investigation. We found that the fluorescence intensity of NDs in injected nude mice were significantly enhanced in a time-dependent manner (Figure 2A,B). Figure 2C,D shows that the fluorescence intensity of NDs in injected nude mice were increased in a concentration-dependent manner after 28 days post-injections. The fluorescence imaging of organs (heart, lung, liver, skin, kidney, and spleen) and skins were dissected on day 28. Most of the NDs are found on the skin comparing with other organs (Figure 2E). Figure 2F shows the comparison of organ weight of NDs-injected nude mice.

### 3.3. Detection of ND-Bearing Lung Cancer Cells In Vitro and Its Tumorigenesis Ability In Vivo

**Figure 3:**
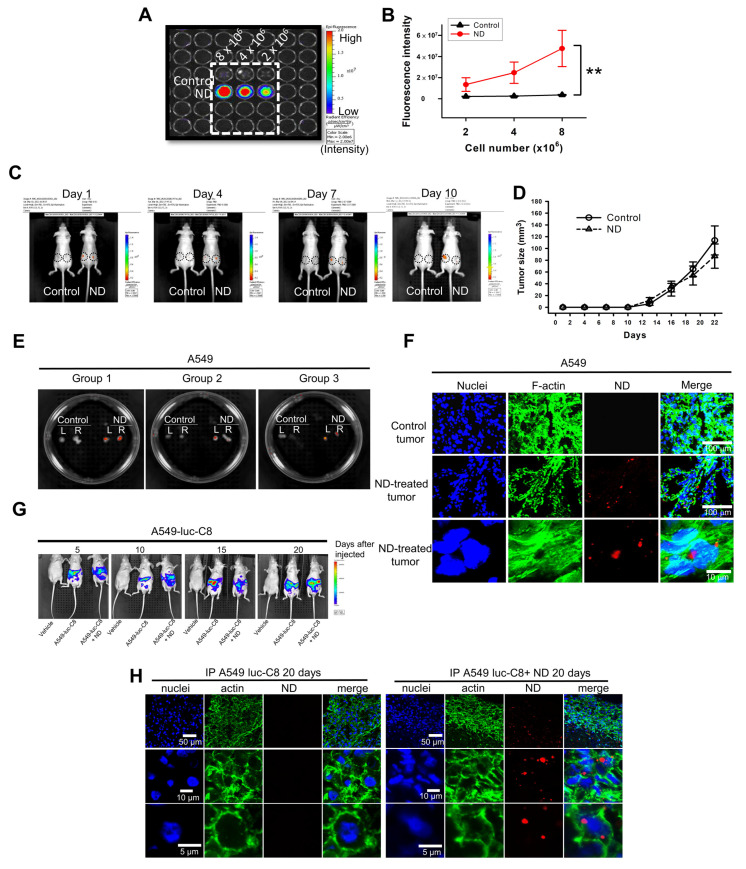
The detection and tumorigenesis of ND-bearing lung cancer cells in vitro and in vivo. (**A**) A549 lung cancer cells were plated at a density of 3 × 10^6^ cells per 100 mm Petri dish for 24 h. Then the cells were incubated with 100 nm ND particles (50 μg/mL for 24 h). At the end of treatment, the ND−treated cells were trypsinized and counted 8 × 10^6^ cells. After centrifugation, the collected cell pellets were resolved in 1×PBS. Then the cells were plated at 96-well plate in serial dilution. The fluorescence intensities of ND−treated cells were observed by IVIS system. (**B**) The fluorescence intensities of ND-treated cells were quantified by IVIS system using Xenogen Living Image^®^ software, Version 4.0 analysis. Results were obtained from three separate experiments and the bar represented the mean ± S.E. ** *p* < 0.05 indicates significant differences compared to the control group. (**C**) 3 × 10^6^ A549 lung cancer ND−treated cells were subcutaneously injected into each hind leg of male BALB/c nude mice (3 weeks old) for 10 days. A549 lung cancer cells for control experiment. The fluorescence intensity was examined by every 3 days. (**D**) Compare the tumor size of ND particles injected subcutaneous into each hind leg of male BALB/c nude mice (3 weeks old) for 22 days. (**E**) Tracking the tumor size with the progression of tumorigenesis in BALB/c nude mice of human A549 lung cancer ND−treated model. After 40 days, all the mice were sacrificed by CO_2_ inhalation, and the tumor section were observed by IVIS. (**F**) The sample were examined under confocal microscope system. The red fluorescence from ND particles was excited with 580 nm, and the emission collected in the range of 600−700 nm. The actin displayed green fluorescence. The nuclei displayed blue fluorescence. (**G**) A549-luc-C8 lung cancer cells were plated at a density of 2 × 10^6^ cells per 100 mm Petri dish for 24 h. Then the cells were incubated with ND particles (50 μg/mL for 24 h). At the end of treatment, the ND-treated cells were trypsinized and counted 1 × 10^7^ cells. Intraperitoneal injection A549-luc-C8 lung into BALB/c nude mice. The tumor formation detected by cell luminescence signals were observed by the IVIS system. (**H**) The sample were examined under confocal microscope system. The red fluorescence from ND particles was excited with 580 nm, and the emission collected in the range of 600–700 nm. The actin displayed green fluorescence. The nuclei displayed blue fluorescence.

The detection of ND-bearing lung cancer cells in vitro was examined by IVIS (Figure 3A). We found that the fluorescence intensity of ND-bearing lung cancer cells was significantly increased based on the cell number counts (Figure 3B). To further investigate the biocompatibility and detection ability of ND in vivo, we subcutaneously injected the A549 and ND-bearing A549 lung cancer cells into BALB/c nude mice to establish xenografted human lung tumors and monitor the tumorigenesis of ND-bearing cancer cells in vivo. The fluorescence signal of ND-bearing lung cancer cells in vivo was observed in nude mice after 10 days (Figure 3C). In addition, ND did not alter tumor size compared with control groups (Figure 3D). After post-injections for 40 days, the treatment groups 1, 2, and 3 were sacrificed and ND red fluorescence in visible tumors was detected by IVIS analysis. Figure 3E shows the xenografted lung tumor tissues. A strong fluorescence signal can be observed from ND-treated groups, whereas no fluorescence signal in control groups. ND particles were located in the tumor tissues separated from nude mice (Figure 3F). These results demonstrate the long-term detection ability of ND and its biocompatibility in lung cancer cells in vitro and in vivo.

We further evaluated the tumorigenesis of ND-bearing lung cancer cells in intraperitoneal tumor. The A549-luc-C8 and ND-treated A549-luc-C8 lung cancer cells were intraperitoneally injected into BALB/c nude mice. Figure 3G shows the detection of luminescence signals upon the tumor formation in the ND-bearing A549-luc-C8 in nude mice. The tissues from A549-luc-C8 and ND-treated A549-luc-C8 lung tumors were separated from the sacrificed mice. NDs were observed in the tumor tissues of nude mice (Figure 3H). We further quantified the red fluorescence intensity of ND particles by ImageJ software. The red fluorescence intensity was increased around 80-fold in the ND-treated group by comparison with the control. Moreover, NDs located in the cytoskeleton of human lung cancer cells were observed in the tumor tissues. These results indicate that ND can be used for monitoring tumorigenesis in lung cancer.

### 3.4. Conjugation of ND and Cetuximab

**Figure 4:**
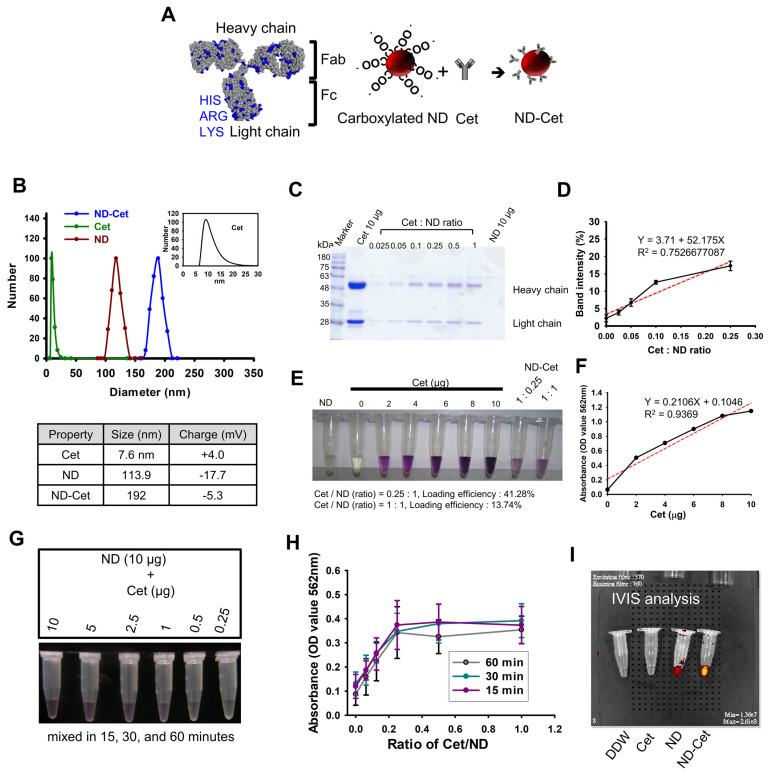
The characterization of the conjugation of ND and Cet. (**A**) The Cet structure shows by PyMOL software. The basic amino acid: arginine (ARG), lysine (LYS), and histidine (HIS) (shown in blue). PI of Cet: 8.44; pH < PI, the protein with positive charge. (**B**) The average size of Cet, ND, and ND-Cet were analyzed by DLS. The concentration of 1 μg/mL of ND and ND-Cet in DDW was used for analysis. The zeta potential of Cet, ND, and ND-Cet were analyzed. The concentration of 0.05 μg/mL of ND and ND-Cet in DDW was used for analysis. The average zeta potentials of particles were calculated as shown in the table. (**C**) Cet (2−10 μg) were mixed with NDs (10 μg). Representative SDS-PAGE data were shown one of three separate experiments with similar findings. (**D**) The quantification of band intensities was calculated for the binding affinity of ND and Cet. (**E**) After being washed by DDW twice, the ND-Cet pellet was re-dispersed by BCA reagent and incubated into 37℃ water bath for 30 min. Finally, we centrifuged to bring the ND to the bottom of the tube and then collect the suspension and assay by ELISA reader. Photograph of the different concentration of Cet (2–10 μg), 10 μg ND, and ND-Cet (ND: Cet = 1:0.25 and 1:1) was shown. (**F**) The OD values was measured and quantified as a standard curve at 562 nm absorbance by ELISA reader. The OD values of ND and Cet (*w*/*w*, 10 μg: 2.5 μg, 10 μg: 10 μg) were compared with stand curve to examine the loading efficiency of Cet were 41.28% for ND: Cet = 1:0.25; 13.74% for ND: Cet = 1:1. (**G**) The requirement of binding time of ND and Cet. The incubation time of ND-Cet at 15, 30, and 60 min were investigated. After being washed by DDW twice, the ND-Cet pellet was re-dispersed by BCA reagent. Finally, we centrifuged to bring the ND to the bottom of the tube and then collect suspension and assay by ELISA reader. Photograph of ND-Cet incubation with 60 min with BCA reagent was shown. (**H**) The OD values of ND-Cet were measured. The data were from 3 independent experiments. (**I**) The fluorescence property of DDW, Cet, ND, and ND-Cet were analyzed by IVIS analysis.

ND particles were modified with carboxyl groups and exhibited high affinity with proteins [32]. ND can non-covalently bound with Cet [17,19,20]. We created the ND-conjugated Cet for the detection and tracking of EGFR in lung cancer cells. Figure 4A shows that Cet interacts with the negative charge surface of carboxylated ND. A negative charge on the NDs surface is attributed to carboxyl groups [33]. Cet contained the basic amino acids including arginine (R), lysine (K), and histidine (H) resulting in a positive charge (blue color region). The zeta potential was applied to measure the average surface charge of Cet, ND, and ND-Cet. The average zeta potential of Cet and ND was approximate at +4.0 mV and −17.7 mV, respectively. The average surface charge of ND-Cet was around −5.3 mV after the conjugation (Figure 4B). The average size distribution of ND-Cet was further analyzed by dynamic laser scattering. The average size of Cet and ND were approximate at 7.6 ± 2.6 nm and 113.9 ± 4.9 nm, respectively. The size of ND-Cet was increased to 192.0 ± 8.1 nm (Figure 4B).

To evaluate the binding affinity of ND and Cet, ND particles were mixed with various ratios of Cet. The centrifuged ND-Cet pellets were analyzed by SDS-PAGE (Figure 4C). The band intensities showed the heavy chain (upper lanes) and light chain (lower lanes) of Cet. The saturated band intensity from Cet /ND ratio was 1, 0.5, and 0.25 (Figure 4D). The proper binding ratio of Cet: ND was about 2.5 μg:10 μg (4:1, *w*/*w*). To further examine the binding affinity, the ND-Cet complexes were analyzed by BCA analysis. The purple color and OD value of ND-Cet complexes were determined by a spectrophotometer (Figure 4E). Based on the standard curve, Cet conjugated with ND was about 41.28% (1.032 μg) when 10 μg of ND mixed with 2.5 μg of Cet (Figure 4F). We also investigated the requirement of binding time of ND-Cet. ND and Cet were incubated with various times and then examined by BCA analysis (Figure 4G). After quantification, various periods of 15, 30, and 60 min did not show significant alteration of binding affinity of ND-Cet (Figure 4H). In addition, NDs conjugated with Cet did not reduce the fluorescence property of NDs (Figure 4I). Cet can be directly adsorbed on the surface of NDs.

### 3.5. Direct Observation of ND-Cet Bind with EGFR Proteins on a Protein G Bead Using Immunoprecipitation and Confocal Microscopy

**Figure 5:**
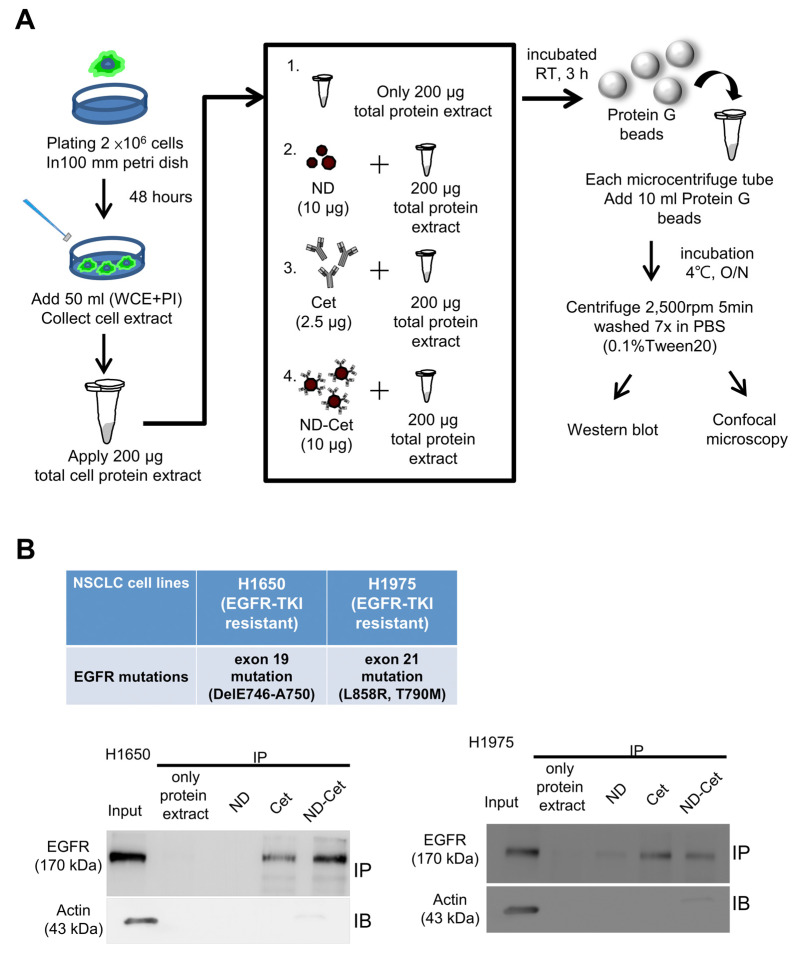
ND-Cet selectively pulled down EGFR proteins of lung cancer cells. (**A**) The immunoprecipitation protocol is to detect EGFR by ND-Cet. The total cellular protein extracts were mixed with Cet (2.5 μg), ND (10 μg), and ND-Cet (10 μg), then incubation at room temperature with continuous mixing for 3 h. The Protein G SepharoseTM beads were added to each sample. (**B**) ND-Cet selectively pulled down EGFR in H1650 and H1975 lung cancer cells with EGFR mutations. Immunoprecipitation of H1650 and H1975 cells using 200 μg cell lysate, 10 μg ND, 2.5 μg Cet, and 10 μg ND-Cet. The EGFR proteins were immunoprecipitated using Cet and ND-Cet. The protein levels were examined by westernblot analysis using specific antibodies. The above representative immunoprecipitation and western blot data were shown from one of three separate experiments with similar findings.

**Figure 6:**
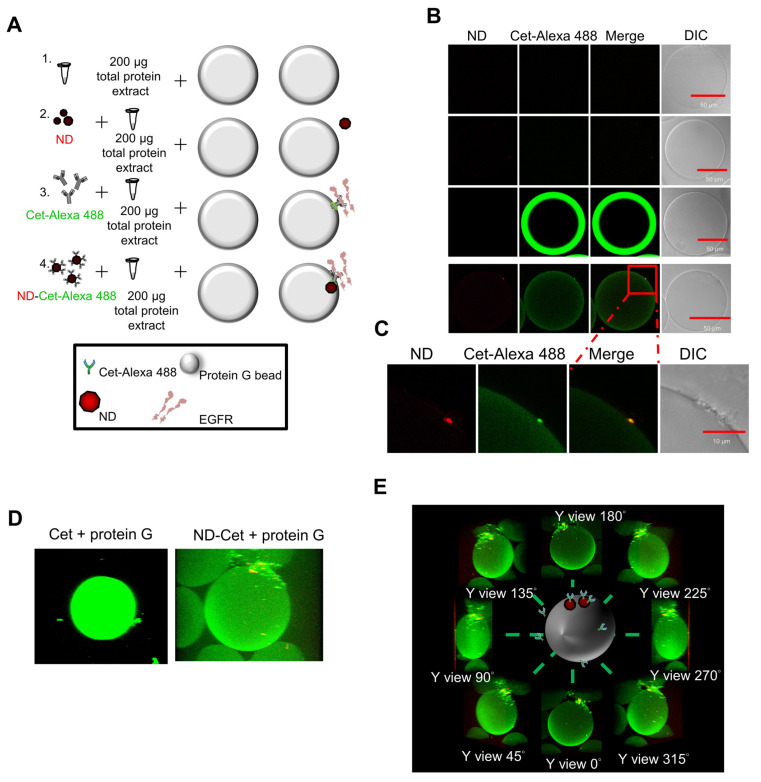
Observation of ND-Cet on a protein G-Sepharose bead binding with EGFR by confocal microscopy. (**A**,**B**) Cet was labeled with a fluorescence dye Alexa 488. The protein G-Sepharose beads were placed on the slide glass and observed under confocal after immunoprecipitation. The green fluorescence of Cet was excited with 496 nm and the emission was collected in the range of 515~520 nm. The red fluorescence of ND was excited with 580 nm, and the emission was collected in the range of 600~700 nm. Representative confocal image data were shown from one of three separate experiments with similar findings. (**C**) The marked red square indicating the edge of protein G bead was amplified. (**D**) Three-dimensional (3D) images show ND-Cet binding with EGFR proteins on protein G-Sepharose beads. The green fluorescence of Cet was excited with 496 nm and the emission was collected in the range of 515~520 nm. The red fluorescence of ND was excited with 580 nm and the emission was collected in the range of 600~700 nm. A protein G bead was from a series of individual Z-slices taken by confocal microscopy. Representative confocal image data were shown from one of three separate experiments with similar findings. (**E**) Different angles-axis rotation of ND-Cet-binding EGFR proteins on a protein G bead.

Figure 5A shows immunoprecipitation to detect the targeting ability of ND-Cet on EGFR in human cancer cells. After immunoprecipitation by ND-Cet, the existence of EGFR on ND-Cet was analyzed by Western blot. ND-Cet can pull down EGFR in H1650 and H1975 lung cancer cells with EGFR mutations (Figure 5B). Actin was used as a loading control. These results demonstrate that the ND-Cet can be applied for the detection of EGFR of lung cancer cells. We further observed the ND-Cet bound with EGFR proteins of A549 cells on a single protein G-Sepharose bead using confocal microscopy (Figure 6A). The protein G-Sepharose bead alone or the bead plus ND did not show any signal of fluorescence (Figure 6B). Cet was labeled with a fluorescence dye alexa488 that showed a bright green-fluorescent ring pattern on the surface of protein G-Sepharose bead (Figure 6B). The green fluorescence of Cet and the red fluorescence of ND were colocalized on a protein G bead (Figure 6B). The marked red square amplified by Figure 6B showed a single ND-Cet bound with EGFR proteins on a protein G-Sepharose bead (Figure 6C). Moreover, the three-dimension (3-D) image of Cet-Alexa 488 and ND-Cet on a protein G bead was shown in Figure 6D and Supplementary videos S1 and S2. Different angles of the rotation of y-axis showed that ND-Cet bound with EGFR proteins on a protein G bead (Figure 6E). It is the first time that ND-Cet complex can be directly observed on the protein G bead to confirm the conjugation of EGFR and Cet after immunoprecipitation process. We have demonstrated that ND-Cet can be applied for the detection of EGFR in vitro by immunoprecipitation and laser scanning confocal microscope. Moreover, we can track the binding ability, location, and final fate of EGFR using ND-Cet.

### 3.6. ND-Cet Selectively Binds to EGFR in Lung Cancer Cells

**Figure 7:**
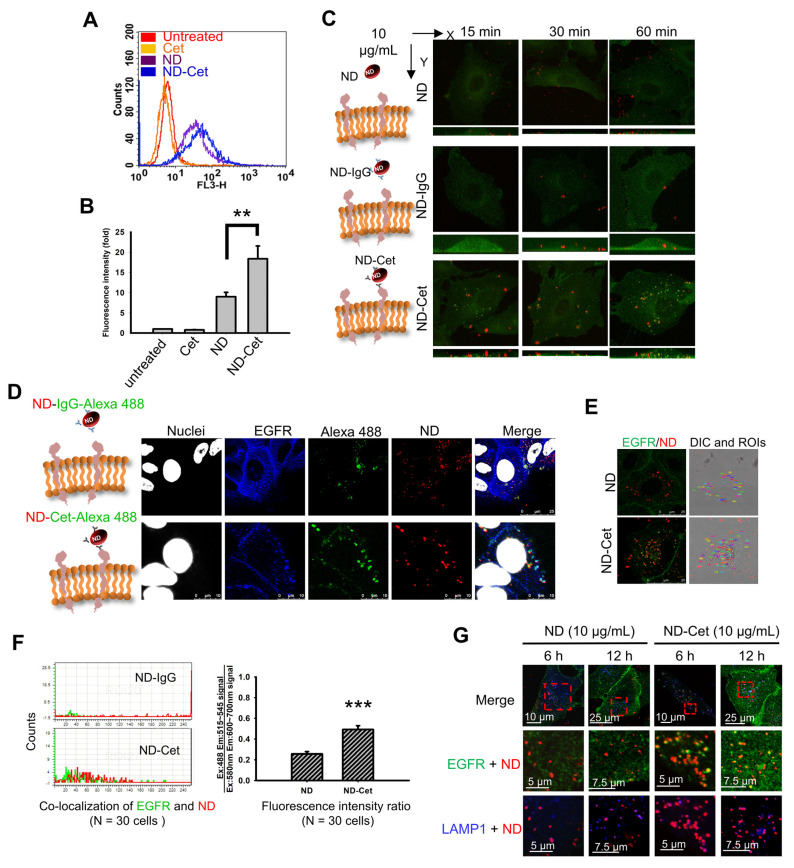
ND-Cet selectively bind EGFR in lung cancer cells. (**A**) Comparison of uptake ability between ND and ND-Cet by flow cytometry. The A549 lung cancer cells were plated at a density of 7 × 10^5^ cells per 60 mm petri. Then the cells were treated with or without Cet (12.5 μg/mL), ND (50 μg/mL), and ND-Cet (50 μg/mL) for 24 h. The fluorescence intensity of ND was excited with wavelength 488 nm, and the emission was collected with >650 nm signal range (FL3-H). (**B**) The data of fluorescence intensity was quantified from a minimum of 10,000 cells using CellQuest software. Results were obtained from three separate experiments and the bar represented the mean ± S.E. ** *p* < 0.01 indicates significant difference between ND treated and ND-Cet-treated cells. (**C**) Detection of ND, ND-IgG, and ND-Cet in short term of time. The cells were treated with 10 μg/mL ND, ND-IgG, and ND-Cet for 15, 30, and 60 min. At the end of treatment, the cells were incubated with rabbit anti-EGFR and then incubated with goat anti-rabbit Hilyte 488 antibody and observed by laser scanning confocal microscope (including z-stacks). The fluorescence intensity of ND was excited with wavelength 580 nm, and the emission was collected in 600~700 nm. The fluorescence intensity of EGFR was excited with wavelength 488 nm, and emission was collected in 515~545 nm. EGFR displays green color. ND displays red color. (**D**) A549 cells were treated with 10 μg/mL ND and ND-Cet for 4 h. At the end of treatment, the cells were incubated with rabbit anti-EGFR antibody and then stained with goat anti-rabbit Hilyte 488 antibody. (**E**) Select ROIs which is ND or ND-Cet inside the cells (cells boundary based on bright field image). (**F**) These ROIs’ pixels from different channels would be investigated. The histogram chart shows that the x-axis is gray values from 0~255, and y-axis is count number of pixels. The mean fluorescence intensity per ROIs in the red and green channel were quantified by Leica LAS AF Lite software of laser scanning confocal microscope. Results were obtained from about 20~30 ND clusters per cell and 30 cells were analyzed for each experiment group. The overlapping coefficients of EGFR/ND were quantified by Leica LAS AF Lite software of laser scanning confocal microscope. The bar represented the mean ± S.E. *** *p* < 0.001 indicates significant difference between ND treated and ND-Cet treated. (**G**) The cells were treated with 10 μg/mL ND and ND-Cet for 6−24 h. At the end of treatment, the cells were co-incubated with rabbit anti-EGFR antibody and mouse anti-LAMP1 then stained with goat anti-rabbit Hilyte 488 antibody and goat anti-mouse Alexa Fluor 405. Green color indicates the location of EGFR. Blue color indicates the location of LAMP1. Red color indicates the location of ND particles. The label was magnified from the marked rectangles. Yellow indicates co-localization of EGFR and ND. Purple color indicates co-localization of LAMP1 and ND.

Nanoparticles can passively target tumor sites via enhanced permeability and retention effects or actively target by ligand conjugations [34]. Active tumor targeting can be achieved by surface functionalization of nanoparticles with ligands such as antibodies, peptides, small molecules, and nucleic acids [35,36]. Cet can induce the EGFR internalization and degradation [37]. To determine whether the ND-Cet selectively targets EGFR, the cellular uptake ability of ND-Cet was evaluated. The cellular uptake ability of A549 cells was relatively higher in ND-Cet than in ND alone (Figure 7A). ND fluorescence intensity in the ND-Cet-treated A549 cells was significantly increased by comparison with ND alone (Figure 7B). ND-Cet interacted with EGFR that increased the internalized levels of EGFR proteins in a time-dependent manner (Figure 7C). ND-Cet-Alexa 488 was selectively bound to EGFR proteins (Figure 7D). To further quantify the fluorescence intensity of EGFR/ND overlapping coefficients between the ND-Cet treated cells and the ND-treated cells. The location of ND inside the cells was selected as the region of interests (ROIs). Each ROIs’ pixels from the green channel (Ex: 488 nm, Em: 515–545 nm) and red channel (Ex: 580 nm Em: 600–700 nm) were recorded. The EGFR/ND overlapping coefficients increased around twice-folds in the ND-Cet treated cells by comparison with the ND treated cells (Figure 7E,F). It has been reported that the conjugation of gold nanoparticle with Cet selectively bind to EGFR, and then induce internalization on EGFR overexpressing glioblastoma cells [38]. We found that ND-Cet may mediate Cet-induced receptor internalization and degradation. These results demonstrate that the conjugation of Cet to NDs can selectively target on the EGFR-expressed cancer cells. We suggest that ND-Cet enhances the uptake ability through the binding of EGFR in cancer cells.

**Figure 8:**
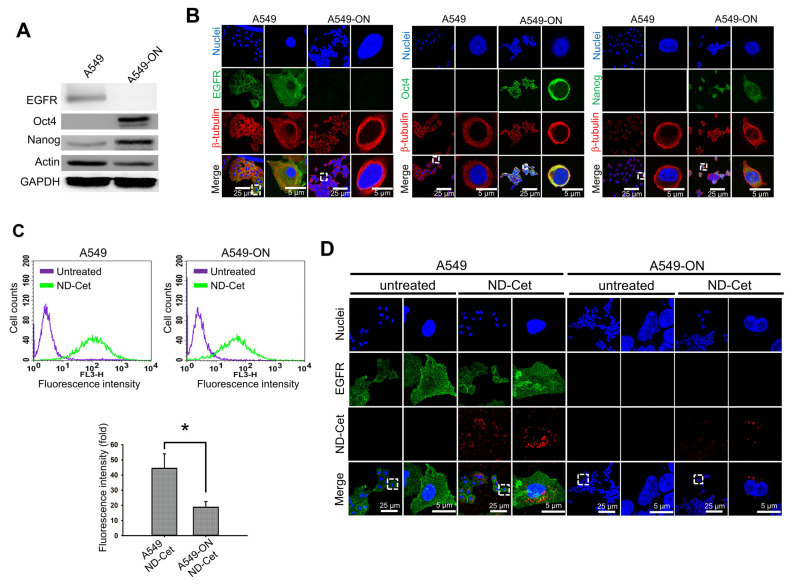

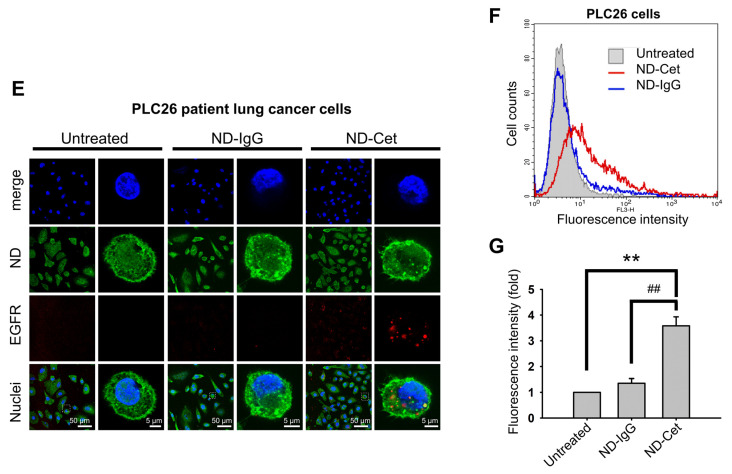
ND-Cet enhances the uptake ability through the selective binding of EGFR. (**A**) The proteins levels of EGFR, Oct4, and Nanog in the A549 and A549-ON cells were analyzed by western blot analysis using specific anti-EGFR, anit-Oct4, anti-Nanog, and anti-Actin (loading control) antibodies, respectively. Representative western blot data were shown from one of three separate experiments with similar findings. (**B**) Representative confocal microscopy images of A549 and A549-ON show the location of EGFR, Oct4, and Nanog proteins. The cells were incubated with rabbit anti-EGFR, anit-Oct4, and anti-Nanog, followed by goat anti-rabbit Dylight 488 antibody. The β-tubulin was stained with Cy3-labeled mouse anti-β-tubulin (red). The nuclei were stained with the Hoechst 33258 (blue). (**C**) A549 or A549-ON cells were treated with or without ND-Cet (10 μg/mL:2.5 μg/mL) for 24 h. At the end of treatment, the cells were subjected to flow cytometry. The fluorescence intensity of NDs was excited with wavelength 488 nm, and the emission was collected with >650 nm signal range (FL3−H). The data of fluorescence intensity were quantified from a minimum of 10,000 cells using CellQuest software. Results were obtained from three separate experiments and the bar represented the mean ± S.E. * *p* < 0.05 indicates significant different between ND-Cet treated A549 and A549-ON cells. (**D**) Intracellular localization of ND-Cet in A549 and A549-ON cells. The cells were treated with or without ND-Cet (10 μg/mL: 2.5 μg/mL) for 24 h. At the end of treatment, the cells were incubated with rabbit anti-EGFR, then stained with goat anti-rabbit Dylight 488 antibody and observed by confocal microscopy analysis. Green color indicates the location of EGFR. Red color indicates the location of ND particles which excited with wavelength 580 nm, and the emission was collected in range from 600 to 700 nm. The nuclei were stained with Hoechst 33258. (**E**) The PLC26 cells were treated with ND-Cet (10 µg/mL) or ND-IgG (10 µg/mL) for 4 h and analyzed by confocal microscope. The green fluorescence from ND particles was excited by a wavelength of 580 nm and the emission was collected in the range 600–700 nm. EGFR and nuclei exhibited green and blue color, respectively. (**F**) The PLC26 cells were plated in 35−mm Petri dish. Then, the cells were treated with ND-Cet (10 µg/mL) or ND-IgG (10 µg/mL) for 4 h. At the end of treatment, the cells were trypsinized and then subjected to flow cytometry. The data of fluorescence intensity was quantified from a minimum of 10,000 cells using CellQuest software. (**G**) The bar represented the mean ± S.E. ** *p* < 0.01 indicates significant difference between untreated and ND-Cet treated PLC26 cells. ## *p* < 0.01 indicates significant difference between ND-IgG and ND-Cet treated PLC26 cells.

To further determine the EGFR specific targeting of NDs, we verify the selective targeting ability of ND-Cet in the A549-ON cells. Oct4 and Nanog were the stem cell markers that control the cancer stem cells fate during cancer development and tumorigenesis [39]. Overexpression of Oct4 and Nanog was associated with advanced cancer stage, decreased rate of patient survival, and acquired chemo-resistance in lung adenocarcinomas [40]. The stem-cell marker Oct4 and Nanog proteins were highly expressed in the A549-ON cells; in contrast, the EGFR protein expressed remarkably in the A549 cells but not expressed in the A549-ON cells (Figure 8A,B). ND-Cet significantly increased the uptake ability of the A549 cells (Figure 8C). Moreover, confocal microscopy images of EGFR positive the A549 cells show colocalization of ND-Cet with EGFR, whereas almost no interaction of ND-Cet with EGFR was observed in EGFR-negative the A549-ON cells (Figure 8D). These results demonstrate that the ND-Cet enhances the uptake ability through the selective binding of EGFR of the A549 lung but not in the A549-ON cells.

### 3.7. ND-Cet Binds Selectively in EGFR of Clinical Patient PLC26 Lung Cancer Cells

We further demonstrate that ND-Cet selectively binds to EGFR in clinical patient PLC26 lung cancer cells. We examined the uptake ability of ND-IgG and ND-Cet in the EGFR-expressed PLC26 lung cancer cells separated from a clinical NSCLC patient. Treatment with ND-Cet (10 μg/mL for 4 h) elevated the location of ND particles inside the cells (Figure 8E). The increased cellular uptake ability of ND-Cet was confirmed by flow cytometry (Figure 8F and G). These results further confirmed that the conjugation of Cet to ND can selectively target EGFR-expressed cancer cells. We suggest that ND-Cet enhances the uptake ability through the binding of EGFR in patient lung cancer cells.

### 3.8. ND-Cet Selectively Binds to EGFR and Internalizes into Lysosome in Lung Cancer Cells

We found that LAMP1 and ND were co-localized after treatment with ND or ND-Cet in a time-dependent manner (Figure 7G). The amplified pictures from the squares that indicated the co-localization of ND or ND-Cet, EGFR, and lysosomes (Figure 7G). Moreover, the LAMP1/ND overlapping coefficients (the binding levels of lysosome and ND particles) were increased both in the ND-treated cells and ND-Cet treated cells (Figure 7G). We quantified the EGFR and lysosome overlapped with ND-Cet. Overlapping coefficient was higher in the ND-Cet-treated cells than ND treated cells.

## 4. Discussion and Conclusions

Recent advances in single-molecule imaging focuses on the nanoprobe with better biocompatibility, brightness (for precise detection), optical stability (for longer detection), specificity, etc. This technique enables direct monitoring of the location, movement, turnover, and functions of biomolecules in living cells. Numerous fluorescent nanoprobes have been developed for biological and biomedical imaging. ND, containing the fluorophores with NV centers, have high photostability and long-lived fluorescence decay lifetime. In this study, we demonstrated the long-term detection of the fluorescence signal of ND in vitro and in vivo. These results reveal that ND can be applied as a long-term tracking agent in vitro and in vivo. Red fluorescence intensity of NDs can be observed in rats and mice with long-term stability and biocompatibility [11]. Currently, a lot of the quantum sensing applications in biology based on ND-containing NVs. It may due to the capability for long-term monitoring the molecular machinery in living systems [41].

Nanoparticles functionalized with specific molecules such as proteins [42], antibodies [43], RNA [44] may modulate pharmacokinetics and targeting recognition, and increase the efficacy of targeted drugs [17,45]. Cet is a specific EGFR antibody for clinical cancer therapy [24]. We manipulate the ND for the detection and tracking of EGFR on human lung cancer cells with non-covalently conjugation of Cet. ND-Cet retains the ability of binding EGFR proteins in vitro and in vivo. We can detect the EGFR proteins of lung cancers by ND-Cet using immunoprecipitation and confocal microscopy. The EGFR proteins can be directly observed on a protein G bead. Our findings provide that the fluorescence ability of ND combined with the targeting ability of Cet antibody can be employed for tracking EGFR and tumorigenesis of cancer cells.

EGFR mutations have been identified as a predictive biomarker for the yield of EGFR-TKIs, such as erlotinib and gefitinib [46,47]. However, EGFR mutations did not disturb the sensitivity of Cet on targeting EGFR [48,49]. A range of EGFR-targeted nanoparticles has been engineered via tethering the Cet [43,50,51]. In this study, ND-Cet can target EGFR in lung cancer with or without EGFR mutations. ND-based drug-delivery system can improve drug retention and treatment efficacy for chemoresistance tumors [52,53,54]. We suggest that ND conjugated with drugs and Cet can specifically bind to EGFR proteins and improve the treatment efficacy in various cancers [17,19,55].

The intracellular uptake of nanoparticles undergoes through endocytosis mechanisms [56,57,58]. ND particles can be through macropinocytosis and clathrin-mediated endocytosis to enter cells [59,60]. However, the rapid accumulation of ND-Cet suggests that ND-Cet uptake occurs via active receptor-mediated endocytosis [61]. It has been shown that Cet bound to EGFR and did not dissociate from the receptor in the endosome [62]. Moreover, the intracellular fate of Cet-induced-internalized EGFR was trafficked to lysosome [61]. In this study, we demonstrated that ND-Cet were bound to EGFR and then co-localized with LAMP1 protein. We can track the EGFR that is transported to the lysosome for EGFR degradation using ND-Cet. We reported that ubiquitin decorated NDs bind to autophagy receptors to form nanoparticulosomes for entry into lysosomes and selective autophagy pathway [63]. The processing of nanoparticles can be through the selective autophagy pathways also called nanoparticulophagy for entering lysosomes [64]. We suggest that the high specificity of ND-Cet binding to EGFR of lung cancer cells and the EGFR proteins can be transported to lysosome for degradation by the nanoparticulophagy pathway.

In conclusion, we demonstrate that the fluorescence signal of ND particles can be real-time detected in the xenografted human lung tumor formation of nude mice. The ND-conjugated specific EGFR antibody can apply for targeting EGFR and monitoring tumorigenesis during lung cancer progression and therapy.

## Data Availability

Not applicable.

## Supplementary Materials

The following supporting information can be downloaded at: https://www.mdpi.com/article/10.3390/pharmaceutics15010111/s1, Video S1: Cet-treated 3D protein G bead; Video S2: ND-Cet-treated 3D protein G bead.
